# Evaluation of the monotherapy effect of crocin on mild-to-moderate diabetic retinopathy: A randomized clinical trial

**DOI:** 10.22038/ajp.2025.26305

**Published:** 2026

**Authors:** Samaneh Sepahi, Mina Mohajeri, Ali Eslami, Seyedeh Maryam Hosseini, Seyed Ahmad Mohajeri

**Affiliations:** 1 *Food and Beverages Safety Research Center, Urmia University of Medical sciences, Urmia, Iran*; 2 *Department of Pharmacodynamics and Toxicology, School of Pharmacy, Mashhad University of Medical Sciences, Mashhad, Iran*; 3 *ye Research Center, Mashhad University of Medical Sciences, Mashhad, Iran*; 4 *Targeted Drug Delivery Research Center, Pharmaceutical Technology Institute, Mashhad University of Medical Sciences, Mashhad, Iran*

**Keywords:** Crocin, Diabetic retinopathy, Best corrected visual acuity Macular thickness, Retinal thickness

## Abstract

**Objective:**

Diabetic retinopathy (DR) is asymptomatic and can lead to severe and irreversible vision loss if not treated promptly. The aim of this study was to evaluate the efficacy of crocin in mild-to-moderate DR patients whose macular center was not involved according to the early treatment diabetic retinopathy study (ETDRS) criteria.

**Materials and Methods:**

Forty patients with primary DR without involvement of the macular center were enrolled and randomized into crocin 15 mg/day and placebo groups for 3 months. At the beginning and 3 months later, best corrected visual acuity (BCVA), fasting blood sugar (FBS) and HbA1c were measured and the thickness of areas around the macula was determined. After three months, the patients were examined by an ophthalmologist and the required specialized tests were performed.

**Results:**

The mean age of the participants was 47-71 years that were 27 males and 13 females. In the intragroup comparison, the thickness around the macula was significantly reduced in the crocin group (p=0.001) compared to placebo group (p=0.67). In comparison between groups, the mean thickness of the areas around the macula at the beginning and end of the study was significantly different (p=0.046). BCVA was not significantly different after 3 months between groups. HbA1C was significantly reduced (p=0.004) in the crocin group. In this study, no specific complication due to drug use was reported.

**Conclusion:**

Based on our findings, crocin was effective in reducing the thickness of the areas around the macula in mild-to-moderate DR. It is suggested that crocin can be considered an effective supplementary drug in preventing the progression and an adjuvant therapy for DR.

## Introduction

Diabetic retinopathy (DR) is one of the most important ocular complications of diabetes. One-third of diabetic patients have retinopathy, and one-third have vision-threatening retinopathy. Most patients with DR, despite having a lesion in the retina, are asymptomatic and the disease can lead to severe and irreversible vision loss in these patients if not treated promptly (Lin et al. 2021). DR is a serious microvascular complication of diabetes that is associated with the destruction of retinal ganglion cells (RGC) (Yang et al. 2017) . Dangerous visual complications are usually caused by increased retinal vascular permeability, retinal or anterior chamber angiogenesis, or destruction of extensive vessels in the central retina (Jampol et al. 2020). Genetic and race are two important risk factors in DR (Cheung et al. 2010). Maturity and pregnancy are known risk factors for DR in patients with type 1 diabetes. Therefore, scheduled retinal examinations should be considered for patients with type 1 diabetes (Olsen et al. 2004; Ziegler et al. 2012). DR is associated with many systemic and lifestyle factors including nephropathy, obesity, alcohol consumption and markers of anemia, hypothyroidism and endothelial dysfunction (Kume and Kashiwagi 2020). 

DR is classified according to its severity scale and clinical features. In non-proliferative diabetic retinopathy (NPDR), there are vascular changes within the retina but no extra-retinal fibrovascular tissue develops. NPDR based on the severity is divided into mild, moderate, or severe. The most advanced level of DR is proliferative diabetic retinopathy (PDR). Diabetic macular edema (DME) that results from abnormal vascular permeability can occur in patients with any severe level of DR (Barth and Helbig 2021; Le et al. 2021). 

The important treatment of DR is anti-VEGF (vascular endothelial growth factor) intravitreal injection. VEGF is a potent stimulant in angiogenesis and vascular permeability, and VEGF inhibitors can block or slow the progression of disease and angiogenesis (Miller 2016) . At present, there is no specific guideline for cases of DME without visual center involvement (mild-to-moderate DME), and physicians recommend control of the underlying disease (optimal control of blood sugar, blood pressure and possibly blood lipids) and lifestyle for these patients (Barth and Helbig 2021; Munk et al. 2022). 

Plant extracts and plant products have various biomedical applications Medicinal plants, either as monotherapy and supplement therapy, have been widely used for the management of various medical conditions (Ghorani‐Azam et al. 2018; Moghadam et al. 2020). Crocus Sativus and its bioactive constituent, crocin, are very valuable herbal supplement with benefits shown in numerous studies (Pitsikas et al. 2008; Sani et al. 2022). Findings from clinical trials demonstrated its efficacy in reducing depression, anxiety, and blood sugar levels in individuals with diabetes (Milajerdi et al. 2018; Tajaddini et al. 2023). In light of this, the research team persisted in their endeavors to examine the efficacy of crocin in addressing the ocular complications experienced by diabetic patients (Ansari-Mohseni et al. 2023; Kolahdooz et al. 2023; Sepahi et al. 2021a; Sepahi et al. 2022).

In a previous study, we showed that the use of crocin at the dose of 15 mg per day can be effective in reducing the central thickness of the macula (CMT) and developing best corrected visual acuity (BCVA) in DME patients. Crocin is the most important active ingredient of saffron, which is found in the form of crocetin glycosides and is the main color pigment in saffron (Sepahi et al. 2018) . Crocin has anti-inflammatory effects and neuroprotective properties, which may contribute in reducing CMT in patients with DME (Sepahi et al. 2018; Sepahi et al. 2021b). 

The aim of this study was to investigate the effect of crocin on mild-to-moderate macular edema without central involvement and whether crocin reduces edema around the fovea, including the perifovea and parafovea.

## Materials and Methods

### Subjects and study design

The study was designed as a double-blind, randomized placebo-controlled clinical trial and performed on patients with mild-to-moderate DR referred to health centers in Mashhad, Iran from August 2018 to July 2022. This study was conducted after approval by Mashhad University of Medical Sciences (Grant No. 970028) and registered to the Iranian Registry of Clinical Trials by with IRCT20130418013058N13 submission code. Patients with mild-to-moderate DR and non-proliferative retinopathy referred to the retina clinic of Khatam Al-Anbia Hospital's ophthalmology center, (Mashhad, Iran) who did not need injection or laser treatment based on the physician's opinion, were included in this clinical trial study after a detailed explanation of the project and obtaining informed consent.

### Inclusion and exclusion criteria

The inclusion criteria were: 1. Patients aged >18 years with type 1 or 2 diabetes without clinically significant macular edema (CSME) according to the ETDRS criteria or non-center involving DME. 2. The distance from the thickening area to the center of the macula should be at least 500 µm, and the thickness of other areas around the macula should be at least 300 µm. 3. BCVA≥ 8/10 according to Snellen chart. 4. Diabetes mellitus with HbA1c between 7% - 8.5 %over the past 3 months. 5. No need to receive standard treatment including injection, macular photocoagulation or pan- retinal laser. 6. Absence of other serious diseases such as liver and kidney failure. 7. The ability to understand the overview of the study, treatment, and possible side effects. 8. The maximum severity of DR should be mild-to-moderate non-proliferative DR without need for laser. 9. Controlled blood pressure.

The severity of DR was graded as mild or moderate based on slit lamp fundus examination by an experienced retinal specialist in comparison with standard photographs of the ETDRS guideline. In NPDR, retinal microvascular lesions, including retinal hemorrhage, microaneurysms, are limited to retina (Group 1991) .

The exclusion criteria were as follows: 1. Diagnosis of other eye abnormalities which cause no improvement in visual acuity despite improvement in macular edema; such as fovea atrophy. 2. Other eye diseases that affect macular edema such as retinal vein occlusion, and uveitis. 3. Patients with anterior eye segment disorders (Cornea, Anterior chamber, Iris). 4. History of vitrectomy surgery. 5. Sub-foveal hard exudates deposition. 6. The presence of PDR that requires treatment. 7. History of surgery or intraocular injection in the past 3 months. 8. Pregnancy or breastfeeding. 9. Vitreous hemorrhage. 10. Center involvement with an increase in thickness of more than 320 μm in the macular center during the study. 11. Receiving standard treatment (Corton, Bevacizumab, or Laser).

### Sample size

The sample size calculation was performed with Sigma plot software version 12.0 (Systat software, Inc., Germany). With standard deviation of 20 and a decrease of at least 25% as a result of the intervention, α = 0.05 and a power of 95%, a minimum sample size of 20 for each group was calculated (crocin and placebo).

### Preparation of crocin and placebo tablet

Saffron stigmas powder was purchased from Saharkhiz Company, Mashhad, Iran. Crocin was obtained from the saffron plant by the crystallization method and using water/ethanol (20:80 V/V), through shaking vigorously, and centrifugation (4000 rpm, 10 min) (Hadizadeh et al. 2010). A mixture of total crocin was extracted but in this study, the term crocin has been used. The pure crocin crystals were obtained at -20°C after 45 days in the dark and washed with acetone to remove impurities. Then, 15 mg of crocin powder with a purity of ˃90% was used to prepare each tablet. Crocin and placebo tablets were prepared the industrial pharmacy lab at the faculty of pharmacy, Mashhad University of Medical Sciences.

### Intervention

Patients with mild-to-moderate DR were divided in to two groups. Patients in one group were given 15 mg crocin tablets daily as monotherapy, and other patients in the placebo group were given one placebo tablet daily for 3 months. At the beginning of the study, the thickness of different macular sections was determined using OCT, and BCVA was measured and recorded in all patients. Finally, after 3 months, the results obtained from the intervention and placebo groups were compared. Also, at the beginning and end of the study, a blood test fasting blood sugar (FBS) and Hemoglobin A1c (HbA1c) level at the clinical laboratory of Qaem hospital (Mashhad, Iran) was performed for all patients.

### Masking and randomization

The tablets were placed in the same shape and size dark colored container and coded by a pharmacist. The patient, treating physician and researcher were not aware of the code of the drugs that were delivered to the patients. So, the distribution of tablets between the two groups was performed randomly.

### Safety

All the participants, both the treatment and control groups, were asked about of any complications and possible side effect (nausea, constipation, etc.). If the patient did not want to continue, based on the suggestion of the specialist, the treatment process and follow-up of the patient would be different and the patient would be excluded from the study. Also, based on the suggestion of treating physician, if the patient needed more serious intervention such as injection of bevacizumab (Avastin) or corticosteroids or laser, they would be excluded from the study.

### Statistical analysis

Data were analyzed by SPSS software version 24. Kolmogorov-Smirnov test was used to check the normality of quantitative data. Quantitative data are described as mean (standard deviation), and qualitative data as frequency. Independent t-test and paired T-test were performed for difference between BCVA, HbA1c, FBS level and the thickness difference of 8 areas around the macula between the groups and within groups. Categorical data and frequency of side effects were compared using Fisher exact test. The variables are reported as mean ± standard error of the mean (SEM). A p less than 0.05 was considered statistically significant.

## Results

### Subject

**Figure 1:**
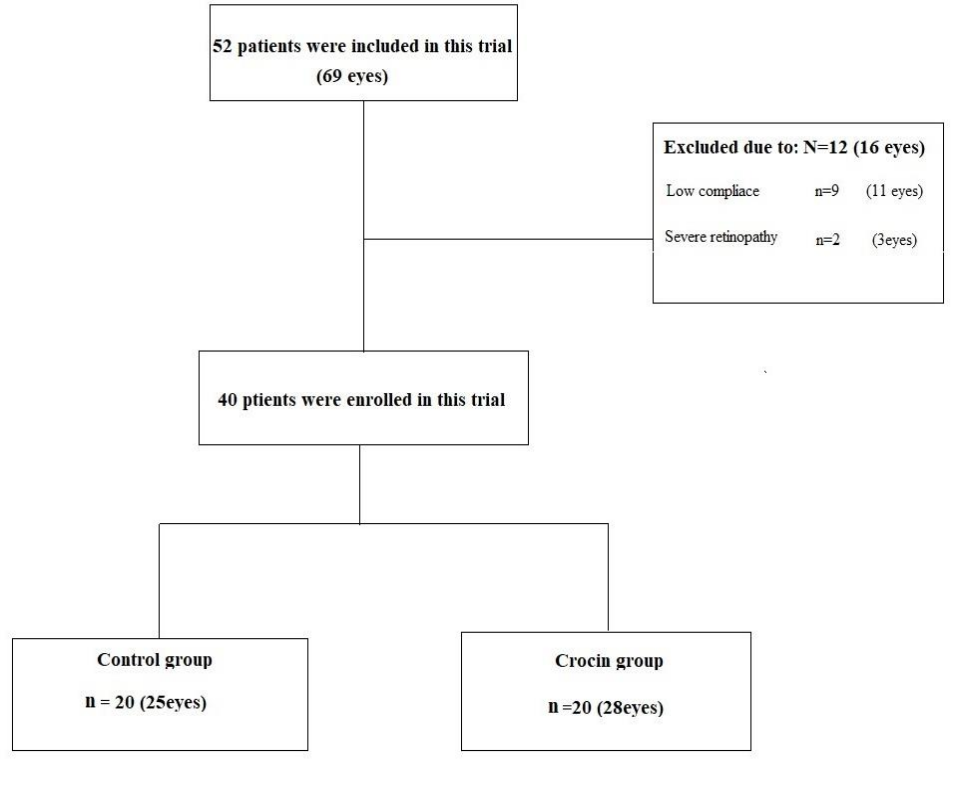
Flowchart illustrating patient inclusion/exclusion and group distribution.

A total of 52 patients (69 eyes) with DR were included in the study, but finally 40 patients (53 eyes) completed the study. Patients were divided into two groups including the treatment group who received 15 mg/day of crocin and the placebo group. Nine patients were excluded due to lack of regular and timely medication, low compliance, and unwillingness to re-cooperate for a second visit. In addition, three patients were excluded due to severe retinopathy and the need for intraocular injection (Figure 1).

### Demographic data

Forty patients completed this trial. In the crocin group, 65% (13) were male and 35% (7) were female with age range of 38-86 years, and in the placebo group, 70% (14) were male and 30% (6) were female with age range of 47-71 years. Age and sex were not significantly different between the two groups. 

Three patients in the placebo group (15%) and 4 in the crocin group (20%) used tobacco and cigarettes (p=0.66). The mean duration of DR was 171.25±1.7 and 201±1.62 months among crocin and placebo groups, respectively (p=0.087) (Table 1).

### Side effect anti-diabetic drugs

One of the researchers made weekly phone calls to patients and possible side effects were asked and recorded. Then, the side effects reported by the patients in crocin and placebo groups were compared (Table 2). In addition, the number of used anti-diabetic drugs is given in Table 3. There was no statistically significant difference in the use of anti-diabetic drugs between the crocin and placebo groups (p>0.05).

**Table 1:** Demographic information of the 40 participants of this Study.

**Variable **	**Crocin (N=20) 15 mg**	**Placebo (N=20)**	**p-value**
Mean Age (year) (mean±SD)	56.42± 2.2	62.14± 1.6	0.62
Sex ratio (Male/Female)	13/7	14/6	0.71
History of diabetes in family (%)	40	50	0.087
Duration of diabetic retinopathy(months) (mean±SD)	171.25±1.7	201±1.62	0.087
Smoking (%)	20%	15%	0. 66

**Table 2:** Frequency of side effects reported in the treatment and placebo groups.

**Side effects**	**Crocin**	**Placebo**	**p-value**
Headache	-	1	0.64
Eye redness	-	1	0.64
Shedding tears	1	1	0.88
Blurred vision	1	-	0.64

**Table 3:** List of drugs used by diabetic retinopathy patients.

**Drug**	**Crocin ** **15mg/day**	**Placebo**	**p-value**
Insulin	18	17	0.86
Metformin	14	16	0.79
Glibenclamide	11	8	0.64
Pioglitazone	3	2	0.86
Gliclazide	17	19	0.78
Sitagliptin	19	18	0.86
Zipmet (Metformin + Sitagliptin)	15	12	0.78
Acarbose	4	3	0.79
Repaglinide	4	6	0.67

### Blood sugar and HbA1c level

Comparison of the mean difference of FBS at the beginning of the study between the crocin and placebo groups did not show statistically significant difference (p=0.07). In addition, three months after the treatment, the mean difference of FBS in the crocin group was significantly less than the placebo group (p=0.014).

**Figure 2:**
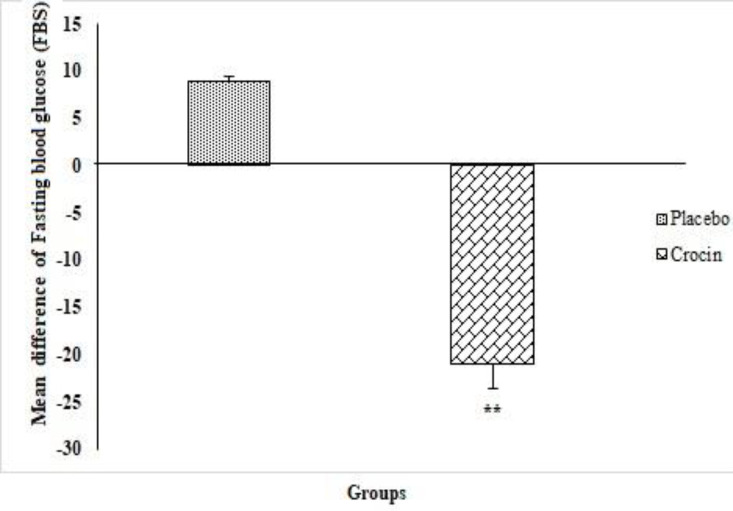
The comparison of FBS mean difference values before and after intervention between the two groups as examined by independent t-test (Mean ± SEM). **p<0.01

Also, in the intra-group comparison, there was a significant difference in FBS in the crocin group before and three months after treatment (p=0.019), but the difference in FBS in the placebo group before and three months after treatment was not significant (p=0.6) (Figure 2). 

**Figure 3:**
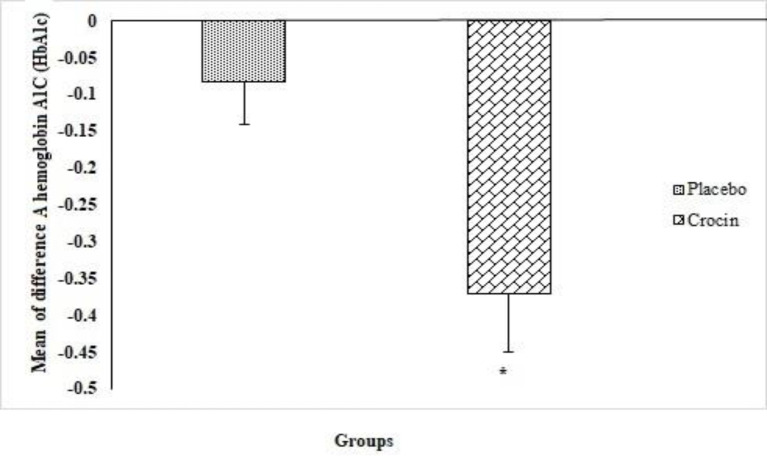
HbA1c mean difference values before and after intervention between the two groups as examined by independent t-test (Mean ± SEM). *p<0.05.

Comparison of the mean difference of HbA1c in the placebo group before and three months after treatment showed no statistically significant difference in the reduction of HbA1c levels in this group (p=0.25). However, comparison within crocin group showed that the reduction of HbA1c level was significant 3 months after the study (p=0.004). In addition, the mean difference of HbA1c between the crocin and placebo groups was statistically significant (p=0.049) (Figure 3).

### BCVA and thickness of area around the macula

**Figure 4:**
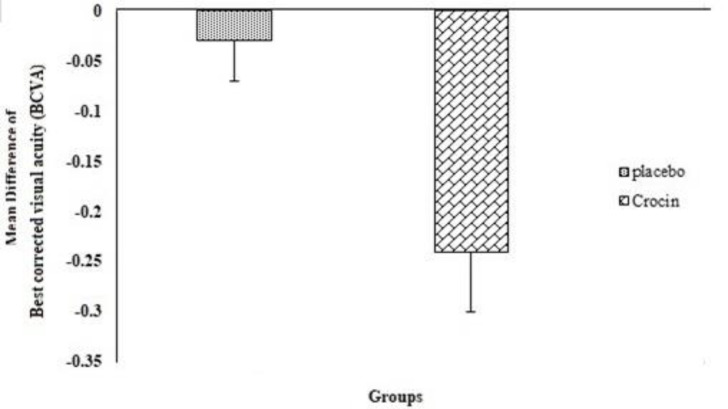
BCVA mean difference values before and after intervention between the two groups as examined by independent t-test (Mean ± SEM).

The difference in BCVA in the placebo (p=0.061) and crocin (p=0.12) groups before and three months after the study was not significant within each group. Also, the mean difference of BCVA between the crocin and placebo groups was not significant (p=0.09) (Figure 4).

The thickness of 8 areas around the macula was measured in all patients with OCT. Examining the difference in the mean thickness of the 8 areas around the macula in the two groups showed that there was a significant was reduced in the mean thickness of these areas in the croicn group than placebo (p=0.046).

**Figure 5:**
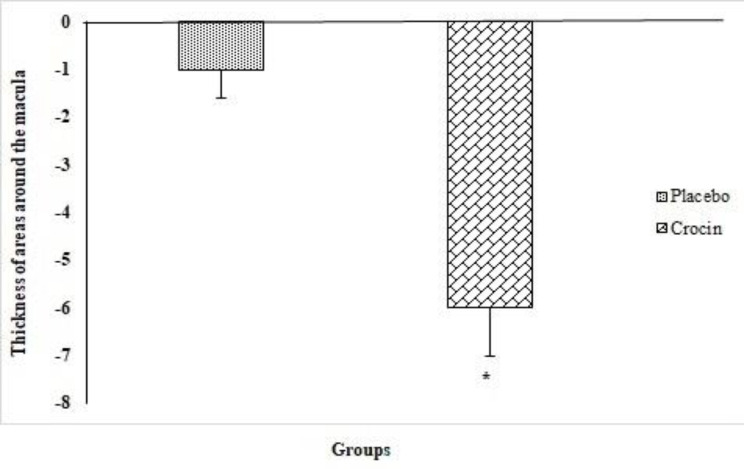
Thickness of areas around the macula mean difference values before and after intervention between the two groups as examined by independent t-test (Mean ± SEM). *p<0.05

The mean difference of thickness in the areas around the macula in the crocin group before and three months after the treatment was significant (p=0.001), but this difference was not significant in placebo group (p=0.67) (Figure 5).

## Discussion

DR is a common complication of diabetes and is the main cause of preventable blindness in people. This disease is detected in one third of people with diabetes and increases the risk of life-threatening systemic vascular complications such as stroke, coronary heart disease and heart failure. Controlling blood sugar, blood fat, and blood pressure is the basis of reducing the risk of developing DR. 

In this study, the effect of crocin was investigated on mild to moderate macular edema in a double-blind, placebo-controlled randomized clinical trial. Our results showed that crocin as a single therapy can significantly reduce thickness of the areas around the macula after 3 months treatment. No significant increase in BCVA was observed in the two groups before and 3 months after study. A significant decrease in HbA1c level in the crocin group was observed before and 3 months after study. In addition, comparison of the HbA1c level between the crocin and placebo groups demonstrated a statistically significant difference. During the study, all patients were in contact with the researchers through phone calls anytime, and were checked bi-weekly for possible side effects of the crocin. Statistical analysis of the reported side effects showed that none of the mentioned side effects were due to the use of crocin or placebo (Table 2).

According to previous studies, crocin tablet at a dose of 15 mg per day does not have any significant side effects for humans (Sepahi et al. 2022). Our previous studies have shown that crocin can be effective in treatments of refractory DME and DR (Ansari-Mohseni et al. 2023; Sepahi et al. 2018), glaucoma (Mahdiani et al. 2023), anxiety and depression (Kazemi et al. 2021; Mohajeri et al. 2020; Talaei et al. 2015) , and diabetes (Sepahi et al. 2022).

In our previous study, retinoprotective effects of crocin and crocetin via anti-angiogenic mechanism in high-glucose-induced human retinal pigment epithelium cells were evaluated. Findings showed that VEGF gene expression and protein level significantly decreased in all treatment groups. In addition, reduction in vascular endothelial growth factor receptor-1 (VEGFR1) gene expression was significantly higher in bevacizumab and crocin + bevacizumab groups than other groups. Only crocin and crocetin could reduce the gene levels of matrix metalloproteinase-2 (MMP-2) and matrix metalloproteinase-9 (MMP-9). In addition, thrombospondin-2 (TSP-2) protein levels increased when HG cells were exposed to crocin or crocin + bevacizumab groups (Sepahi et al. 2021b).

In a clinical study, 68 patients with refractory DME were divided into three groups: crocin 5 mg, crocin 15 mg and placebo. HDL (high-density lipoprotein), LDL (low-density lipoprotein), cholesterol, triglycerides, FBS (fasting blood sugar), HbA1c (hemoglobin A1c), AST (aspartate aminotransferase), ALT (alanine aminotransferase), and BUN (blood urea nitrogen), creatinine, calcium, phosphorus, sodium and potassium were checked in all patients before and 3 months after the intervention. The mean reduction of CMT in the crocin 15 mg group was 82 (p=0.05) and in crocin 5 mg was 41 (p=0.09) compared to the control group. In this study, it was found that the consumption of crocin with a dose of 15 mg has a positive effect on the treatment of macular edema and it can reduce the thickness of the central macula and improve BCVA (Sepahi et al. 2018).

In a study by Xuan and et al., the impact of crocin and its analogues on the blood flow in the retina of rabbits, as well as the functioning of the rat retina was performed (Xuan et al. 1999). The measurement of rabbit retinal blood flow was carried out utilizing colored microspheres, while the assessment of rat retinal function entailed the utilization of electroretinogram methods. The researchers involved in this investigation assert that while there exist numerous compounds capable of enhancing blood flow in the eye, none of them possess the specificity to augment retinal and choroidal blood flow. This unique capability is exclusive to crocin and its analogues. Consequently, it can be concluded that crocin and its analogues serve as specific compounds to heighten retinal blood flow and expedite the recovery of nerve tissue (Xuan et al. 1999).

Another study investigated the effect of saffron plant extract on eye pressure in patients with open-angle glaucoma. In this study, the patients were divided into two groups (saffron and placebo), IOP was still significantly lower in the saffron group after 4 weeks (p=0.001). (Jabbarpoor Bonyadi et al. 2014). 

A study was conducted by Broadhead and colleagues in 2019 to examine the impact of saffron on age-related macular degeneration (AMD). This investigation employed a double-blind, placebo-controlled design, wherein a cohort of over 100 individuals, aged 50 years and above, was selected. Specifically, 50 participants were assigned to the saffron group, while the remaining 50 were assigned to the placebo group. Both groups consumed their respective treatments for a duration of 3 months. Following this initial phase, the medications were swapped between the groups, such that the saffron group now received the placebo and vice versa. Subsequently, after another 3-month period, relevant parameters related to AMD, including BCVA, were measured. The findings of this study indicate a statistically significant increase in BCVA among patients receiving saffron therapy compared to those receiving the placebo (Broadhead et al. 2019).

The result showed that crocin can be effective in reducing the thickness of retina, which can help to prevent the progression of the disease. Also, in severe cases, it can help to treat the disease along with the standard treatment in patients with no serious side effects (Sepahi et al. 2018). According to this studies, crocin can be used in patients with retinopathy as a single therapy in mild cases and as a supplement in severe cases. Therefore, the present clinical trial study aimed to investigate the effect of crocin on mild-to-moderate DR. Considering the prevalence and frequency of DR in the country as a serious complication of diabetes and the aggressiveness of treatment methods for the side effects of drugs used in these patients, finding an effective complementary treatment in this field can be an effective step in solving the patients' problems and their recovery process. In addition to positive effects, herbal medicines have very few side effects. As a result, this study proved the effect of crocin in reducing the thickness of the areas around the macula in mild-to-moderate DR. It is suggested that crocin can be used as an effective drug supplement in preventing the progression and adjunctive treatment of diabetic maculopathy.
